# New Medical Journals

**Published:** 1851-02

**Authors:** 


*New Medical Journals.*—Two new Medical Journals have recently made their appearance.

The *Philadelphia Lancet,* is to be issued semi-monthly, at one dollar per annum. It is a folio with double columns, and is intended to be a medical newspaper. The Editor, Thomas Dunn English, M. D., states, in his salutatory that he shall "oppose quackery in any and every place;" that he shall oppose all false reverence for men and cliques, adding that "we have no reverence for any opinions contrary to our own;" and that his primary aim is an "aggregation of useful facts." The Editor enters upon his undertaking with sanguine expectations. He says "that we shall succeed we feel confident, for we laid the proper foundation to our fabric before we raised the superstructure." Firm faith in success doubtless contributes to its attainment, and we hope that, if necessary, it may prove so in this instance.

The *Stethoscope; and Virginia Medical Gazette* is the title of a new journal, issued at Richmond, Va. This is a monthly of 64 pages, we l gotten up, at $3 per annum, or $4 at the end of the year. By the cognomen selected we infer the objects of the Editor, Dr. P. Clairbone Gooch, to be to explore the internal, vital elements of science and the profession, and to study the *signs* of the times. We cordially wish him success in his diagnostic efforts, and trust he may succeed in curing the maladies which his investigations may discover.

